# Solid Fuel, Secondhand Smoke, and Lung Cancer Mortality: A Prospective Cohort of 323,794 Chinese Never-Smokers

**DOI:** 10.1164/rccm.202201-0114OC

**Published:** 2022-05-26

**Authors:** Elvin S. Cheng, Ka Hung Chan, Marianne Weber, Julia Steinberg, Jane Young, Karen Canfell, Xue Qin Yu

**Affiliations:** ^1^ Sydney School of Public Health and; ^2^ the Daffodil Centre, the University of Sydney, Sydney, New South Wales, Australia;; ^3^ Oxford British Heart Foundation Centre of Research Excellence and; ^4^ Clinical Trial Service Unit and Epidemiological Studies Unit, Nuffield Department of Population Health, University of Oxford, Oxford, United Kingdom; and; ^5^ Prince of Wales Clinical School, University of New South Wales, Sydney, New South Wales, Australia

**Keywords:** lung neoplasms, indoor air pollution, tobacco smoke pollution, nonsmokers

## Abstract

**Rationale:**

Household air pollution and secondhand tobacco smoke are known carcinogens for lung cancer, but large-scale estimates of the relationship with lung cancer mortality are lacking.

**Objectives:**

Using the large-scale cohort China Kadoorie Biobank, we prospectively investigated associations between these two risk factors and lung cancer death among never-smokers.

**Methods:**

The Biobank recruited 512,715 adults aged 30–79 years from 10 regions in China during 2004–2008. Self-reported never-smoking participants were followed up to December 31, 2016, with linkage to mortality data. Total duration of exposure to household air pollution was calculated from self-reported domestic solid fuel use. Exposure to secondhand tobacco smoke was ascertained using exposure at home and/or other places. Hazard ratios and 95% confidence intervals for associations between these two exposures and lung cancer death were estimated using Cox regression, adjusting for key confounders.

**Measurements and Main Results:**

There were 979 lung cancer deaths among 323,794 never-smoking participants without a previous cancer diagnosis during 10.2 years of follow-up. There was a log-linear positive association between exposure to household air pollution and lung cancer death, with a 4% increased risk per 5-year increment of exposure (hazard ratio = 1.04; 95% confidence interval = 1.01–1.06; *P* trend = 0.0034), and participants with 40.1–50.0 years of exposure had the highest risk compared with the never-exposed (hazard ratio = 1.53; 95% confidence interval = 1.13–2.07). The association was largely consistent across various subgroups. No significant association was found between secondhand smoke and lung cancer death.

**Conclusions:**

This cohort study provides new prospective evidence suggesting that domestic solid fuel use is associated with lung cancer death among never-smokers.

**At a Glance Commentary**

**Scientific Knowledge on the Subject**

Household air pollution (HAP) and second-hand tobacco smoke (SHS) have been classified as major risk factors for lung cancer among never-smokers. However, existing evidence on the association of HAP with lung cancer risk has primarily come from case-control studies where temporality cannot be established. Also, evidence based on previous cohort studies was limited as they were retrospective in design, confined to specific geographical regions, or of small sample size. Evidence on the association between SHS and lung cancer risk also has inconsistencies between case-control and cohort studies, with cohort studies tended to report a null association. This warrants reflection on the limitations due to small sample size and/or potential recall bias in previous studies.

**What This Study Adds to the Field**

This large-scale prospective study provides evidence to suggest that HAP exposure as indicated by cumulative exposure to domestic solid fuel use was positively associated with lung cancer death in a dose-response manner, and there is robust evidence to support temporality, for which previous studies had difficulty to establish. This adds new prospective evidence to the literature on the potential carcinogenicity of HAP from domestic solid fuel use across the world. In contrast, prospective association between SHS and lung cancer death was not found in this study.

Lung cancer is the leading cause of cancer-related deaths globally and in China (1). Of the 1.8 million lung cancer deaths globally in 2020 (1), more than one-third occurred in China, where lung cancer mortality has been projected to rise even further (2). Tobacco smoking has long been known to be the leading risk factor for lung cancer, and household air pollution (HAP) and secondhand tobacco smoke (SHS) have been proposed as major risk factors for lung cancer in never-smokers (3).

HAP is predominantly caused by incomplete combustion of solid fuels, which include mainly coal and biomass (e.g., wood and charcoal) used for domestic cooking and/or heating (4). Despite the gradual decline in use of solid fuels worldwide, it is estimated that about half of the world’s population (3.8 billion people) were affected in 2019, and more than 450 million individuals in China still rely on solid fuels (4). SHS is also a common source of air pollution and a major public health problem globally. As the world’s largest producer and consumer of tobacco, China has more than 315 million people who smoke (5). Up to 72% of the Chinese population were exposed to SHS depending on age, sex, and region of residence (6), and about 16% of lung cancer in never-smokers is potentially attributable to SHS (7).

The International Agency for Research on Cancer (IARC) has classified both emissions from indoor combustion of coal and SHS as known human carcinogens (8, 9). However, evidence on the risk of lung cancer in relation to HAP has primarily come from case-control studies, with relatively small sample sizes, limited geographical diversity within study, and marked heterogeneity across studies. Also, the few existing cohort studies on HAP used retrospectively collected data (10–12), and among several large cohort studies on SHS (13–16), some have reported no association between SHS and lung cancer risk (14, 15). Therefore, more evidence from prospective cohort studies for IARC’s conclusions is warranted. Using the China Kadoorie Biobank (CKB), a large prospective cohort study, we examined associations between solid fuel use, SHS exposure, and lung cancer mortality among never-smokers. Some results of this study have been previously reported as a conference abstract (17).

## Methods

### Study Design and Population

Details of the design and methods of the CKB have been described elsewhere (18). Briefly, 512,715 adults (59% women) aged 30–79 years were recruited during 2004–2008 across 10 geographically defined regions (5 urban and 5 rural) in China. At baseline, each eligible participant provided written informed consent, completed an interviewer-administered electronic questionnaire on sociodemographic, lifestyle, environmental factors and biomedical information, and underwent a range of physical measurements (details at http://www.ckbiobank.org) (18). After completion of the baseline survey, a resurvey of a randomly selected sample (about 4%) of the surviving participants was undertaken during 2008. The CKB was approved by the ethics committees of the University of Oxford and the China National Center for Disease Control and Prevention.

The present study focused on 327,138 never-smokers as defined by the International Consortium for Health Outcomes Measurement (19) (i.e., those participants responded “no” to the baseline question, “In your lifetime, have you smoked a total of at least 100 cigarettes or equivalent?” After excluding 1,724 participants who had a self-reported physician diagnosis of cancer at baseline, and 1,620 with potentially unreliable information indicated by the recalled duration of living together with a person who smoked or the total duration of all their three residences exceeding the age at baseline, 323,794 participants remained for the main analysis.

### Ascertainment of Household Air Pollution and Secondhand Smoke

At baseline, participants reported detailed exposure to HAP in their three most recent residences and the duration lived in each residence. Information collected included cooking frequency (daily, weekly, monthly, never or rarely, or no cooking facility) and primary fuel type used (gas, coal, wood, electricity, or other), stove slow-burning frequency (yes always, yes sometimes, or no) and fuel type (smokeless coal, smoky coal, coal brick or coalite, or other), and availability of ventilation facilities (yes, not all stoves, or no). Slow-burning stoves (*see* pictures in Figure E1 in the online supplement) are a special type of stove that burn coal or coal bricks continuously at relatively low temperatures during non-cooking times and are commonly used in coal-reliant regions of China. Participants also reported the period (in calendar years) when the interior of their residence was “coal-smoky” in winter (i.e., with visible or smellable air pollution generated from the burning coal for heating).

A composite proxy of cumulative exposure to HAP was derived from the sum of duration of exposure to *1*) the use of solid fuels for cooking, *2*) use of smoky coal in slow-burning stoves across three residences, and *3*) a coal-smoky home (i.e., smoky inside the house caused by burning coal) during winter in participants’ lifetime (18). The aggregated duration of solid fuel use for cooking was calculated with a weighting of 1.0 for those who reported cooking daily or 0.5 for cooking weekly (20). The aggregated duration of smoky coal use in slow-burning stoves was calculated with a weighting of 1.0 for those who reported having the stove always on and 0.5 for those with the stove sometimes on. The aggregated duration of exposure to a coal-smoky home in winter was calculated by multiplying the reported duration of this with a region-specific weighting coefficient based on the number of months with an average temperature under 10°C during 1999–2017, a proxy for regional winter intensity and the need for heating (21) (Appendix E2). The variables for cumulative duration of HAP exposure were then defined as *1*) a categorical variable of ever versus never exposure to HAP; *2*) a continuous variable for exposure duration; and *3*) a categorical variable for exposure (i.e., never exposed, 0–10, 10–20, 20–30, 30–40, 40–50, or >50 yr).

Exposure to SHS was analyzed as *1*) a categorical variable of exposure for more than 6 months at home (never exposed; past exposed; exposed at baseline); *2*) a continuous variable for the number of years of living with a person who smoked, among those who were exposed at home at baseline; *3*) a categorical variable of ever or never exposure at home, workplace, or in public places; and *4*) a combined variable of the “duration of exposure at home” and “frequency and/or duration of exposure at baseline”: “never lived with a person who smoked” and “never or occasionally exposed at baseline” (reference group); “never lived with a person who smoked” or “never or occasionally exposed at baseline” (group 1); “ever lived with a person who smoked for ⩽30 years” and “exposed at least 1 day per week at baseline” (group 2); “ever lived with a person who smoked for >30 years” and “exposed <7 hours per week at baseline” (group 3); and “ever lived with a person who smoked for >30 years” and “exposed ⩾7 hours per week at baseline” (group 4). Full details for the ascertainment of HAP and SHS exposure variables are described in Appendix E2 and E3, respectively.

Covariate information collected at baseline included a range of sociodemographic, environmental, biomedical, and lifestyle factors. Full details on covariate assessment are described in Table E1.

### Ascertainment of Outcome Measure

The primary outcome of our study was death from lung cancer (International Classification of Diseases, 10th Revision code C34). Information on lung cancer and all-cause mortality was collected regularly from baseline until December 2016, through linkages, via a unique national identification number, with China’s Disease Surveillance Points system, which provided mortality statistics for the entire country (18). It was supplemented by health insurance database information and annual active validation of survival using local residential and administrative records. Fatal events were documented by Disease Surveillance Points coders blinded to baseline information using the International Classification of Diseases, 10th Revision (22).

#### Statistical analyses

Baseline characteristics of the study sample were described as means and SDs, or percentages by categories. The reproducibility of the reported HAP and SHS exposure data was assessed using kappa statistics (23) for the subsample with complete exposure information at baseline and resurvey. Multivariable Cox proportional hazards regression, with age as the underlying time scale (i.e., baseline age as the start of follow-up time), was used to estimate adjusted hazard ratios (HRs) and 95% confidence intervals (CIs) for the association between exposures and lung cancer death. The covariates used for adjustment were selected based on both evidence from prior studies and the statistical or biological potential to act as confounders in the relationship between HAP and/or SHS and lung cancer mortality, and as listed in Table E1, they included sex (male; female), height (continuous), highest level of education (“no formal school or primary school”; ”middle school or high school”; ”technical school or college or university”), occupation at baseline (“agriculture and related workers or factory worker”/others), region of residence, stove ventilation (“no ventilation in all three residences”; ”ventilation in any of the three residences”), solid fuels for heating, use of cooking oil (“peanut or other”; ”rapeseed, soybean, or lard oil”), physical activity (continuous), body mass index (continuous), diabetes (no; yes), intake of dairy products (never or rarely; monthly; 1–6 days weekly; daily), and mutual adjustment for HAP and SHS indicators (3). All participants were followed up from baseline to the date of death, or censored upon death due to other causes, loss to follow-up (1.0%), or to the end of the study period (December 31, 2016), whichever occurred first. Given the known geographical variation of HAP exposure, we conducted stratified analyses by study regions and meta-analyzed the results to compare with the main analysis. Also, subgroup analyses were conducted by sex, age, area of residence (rural; urban), history of respiratory diseases, and self-rated health status. We further tested for an interaction between HAP (as continuous variable) and SHS (as a categorical variable for more than 6 months’ exposure at home: never exposed; past exposed; exposed at baseline), which was tested on the multiplicative scale. In addition, we conducted two sensitivity analyses, first using the longest duration of exposure among the three main sources instead of the sum of duration to provide a lower bound of the estimated duration of HAP exposure, and second, excluding data in the first 5 years of follow-up (i.e., to start the follow-up analysis from the 6th year onwards and exclude any participants who died earlier) to evaluate risk of reverse causality or bias from subclinical lung cancer at baseline. The proportional hazards assumption in the Cox regression models was assessed using Schoenfeld residuals (24). All statistical analyses were performed in SAS software version 9.4, and significance was defined as *P* < 0.05.

## Results

The mean age of the 323,794 never-smoking participants (89% women) was 51.5 (SD = 10.6) years. At baseline, 84.8% of these participants were ever exposed to HAP and 91.0% ever exposed to SHS, with 78.2% ever exposed to both HAP and SHS and only 2.4% never exposed to either. Among participants ever exposed to HAP, solid fuel use for cooking (prevalence 67%) was the most common HAP source, followed by a coal-smoky home in winter (prevalence 27%). Compared with the never-exposed, participants exposed to HAP were more likely to be older, female, from rural regions, less educated, without kitchen ventilation, using lard, soybean, or rapeseed cooking oil, and less physically active, have ever lived with a person who smoked at home, and consume fewer dairy products. Conversely, participants ever exposed to SHS were more likely to be younger, more physically active, and more exposed to cooking solid fuels, stove smoky coal, or a coal-smoky home during winter than the never-exposed group (*see* Table 1). When stratified by sex, HAP exposure among men was mainly from a coal-smoky home in winter, rather than from cooking solid fuel use, as in women (Table E2). Consistent responses were observed across baseline and resurvey, where 83.9–88.7% of the 12,381 never-smokers included in the resurvey reported consistent responses (*see* Table E3).

**Table 1.** Baseline Characteristics of Study Participants Exposed to Household Air Pollution and Secondhand Tobacco Smoke in China Kadoorie Biobank (2004–2008)

	Exposure to Household Air Pollution							Exposure to Secondhand Tobacco Smoke				
Characteristics	All Participants (*n* = *323,794*)		Never Exposed (*n* = *49,236*)		Ever Exposed (*n* = *274,558*)		*P* Value*	Never Exposed (*n* = *29,245*)		Ever Exposed (*n* = *294,549*)		*P* Value*
Proportion of all participants, %	100		15.2		84.8		—	9.0		91.0		—
Mean age at baseline (SD), yr	51.5 (10.6)		48.9 (10.5)		51.9 (10.5)		<0.0001	55.0 (11.2)		51.1 (10.4)		<0.0001
Mean height at baseline (SD), cm	155.5 (6.9)		157.6 (6.9)		155.1 (6.9)		<0.0001	156.6 (7.6)		155.4 (6.9)		<0.0001
Women, %	88.7		78.0		90.7		<0.0001	80.6		89.5		<0.0001
Rural, %	54.0		27.7		58.7		<0.0001	34.2		55.9		<0.0001
Primary school or lower, %	53.4		33.0		57.1		<0.0001	49.0		53.8		<0.0001
Agricultural or factory worker, %	51.1		34.2		54.2		<0.0001	35.8		52.7		<0.0001
No ventilation in all three residences, %	14.1		3.9		15.9		<0.0001	9.0		14.6		<0.0001
Use of lard, soybean, or rapeseed cooking oil in all three residences, %	22.3		5.6		25.3		<0.0001	17.6		22.8		<0.0001
Normal BMI (22.0–25.0 kg/m^2^)^†^, %	38.4		41.1		38.0		<0.0001	36.7		38.6		<0.0001
Mean BMI (SD), kg/m^2^	23.8 (3.4)		23.6 (3.2)		23.9 (3.5)		<0.0001	23.9 (3.5)		23.8 (3.4)		<0.0001
Mean physical activity (SD), MET-h/d	20.5 (13.0)		21.2 (13.8)		20.4 (12.9)		<0.0001	18.1 (13.0)		20.7 (13.0)		<0.0001
Mean cooking solid fuel use (SD), years	16.3 (15.4)		0		19.3 (15.0)		<0.0001	11.9 (15.4)		16.8 (15.4)		<0.0001
Mean slow-burning stove smoky coal use (SD), years	1.5 (5.4)		0		1.7 (5.8)		<0.0001	1.3 (5.4)		1.5 (5.4)		<0.0001
Mean coal-smoky home in winter (SD), years	6.6 (8.1)		0		7.8 (8.2)		<0.0001	6.3 (9.5)		6.7 (8.0)		<0.0001
Ever lived with a person who smoked at home for ⩾6 mo, %	79.6		65.7		82.1		<0.0001	0		87.5		<0.0001
DM, %	2.8		2.6		2.9		0.0001	4.1		2.7		<0.0001
Daily dairy intake, %	10.6		13.3		10.1		<0.0001	16.2		10.1		<0.0001
Study region, *n* (%)												
Qingdao	22718	(7.0)	3046	(6.2)	19672	(7.2)	—	5359	(18.3)	17359	(5.9)	—
Harbin	36470	(11.3)	1928	(3.9)	34542	(12.6)	—	3532	(12.1)	32938	(11.2)	—
Haikou	22783	(7.0)	12699	(25.8)	10084	(3.7)	—	5686	(19.4)	17097	(5.8)	—
Suzhou	32622	(10.1)	5121	(10.4)	27501	(10.0)	—	1393	(4.8)	31229	(10.6)	—
Liuzhou	34458	(10.6)	12791	(26.0)	21667	(7.9)	—	3264	(11.2)	31194	(10.6)	—
Sichuan	31513	(9.7)	1339	(2.7)	30174	(11.0)	—	839	(2.9)	30674	(10.4)	—
Gansu	32138	(9.9)	825	(1.7)	31313	(11.4)	—	540	(1.9)	31598	(10.7)	—
Henan	39652	(12.3)	4	(0.0)	39648	(14.4)	—	1005	(3.4)	38647	(13.1)	—
Zhejiang	35490	(11.0)	11331	(23.0)	24159	(8.8)	—	6299	(21.5)	29191	(9.9)	—
Hunan	35950	(11.1)	152	(0.3)	35798	(13.0)	—	1328	(4.5)	34622	(11.8)	—

*Definition of abbreviations*: BMI = body mass index; DM = diabetes; MET = metabolic equivalent of task.

* *χ*2 tests were used when comparing categorical variables and two-sample *t* tests were used when comparing continuous variables.

^†^ Using the classification by Wang and colleagues (45).

**Figure 1.**
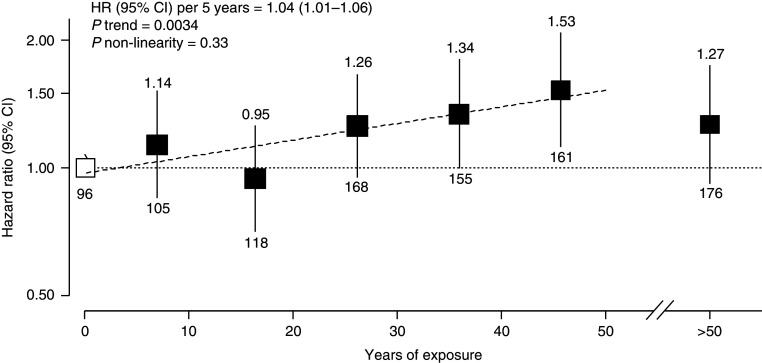
Adjusted hazard ratios (HRs) and 95% confidence intervals (CIs) for lung cancer deaths among never-smokers by years of exposure to household air pollution in China Kadoorie Biobank (2004–2008). HRs were calculated from multivariable analyses with baseline age as the underlying time variable and adjusted for sex, height, region of residence, highest education, occupation, stove ventilation, solid fuel use for heating, physical activity, body mass index, diabetes, and intake of dairy products, using cooking oil use as a stratified variable. The black squares represent HRs corresponding to participants in each group of the exposure categories (i.e., 0–10, 10–20, 20–30, 30–40, 40–50, or >50 yr) with the size proportional to the inverse variance of each effect size. The vertical lines represent 95% CIs. The numbers above the vertical lines are point estimates for HRs, and the numbers below the lines are numbers of events. The dashed line represents the slope from a weighted linear regression in the range of 0–50 years of exposure, with weights based on the inverse variance of the log HRs, and the last group (>50 yr) was excluded from both the trend test and test for nonlinearity, as well as for calculating the estimated risk in HR (95% CI) per 5 years.

**Figure 2.**
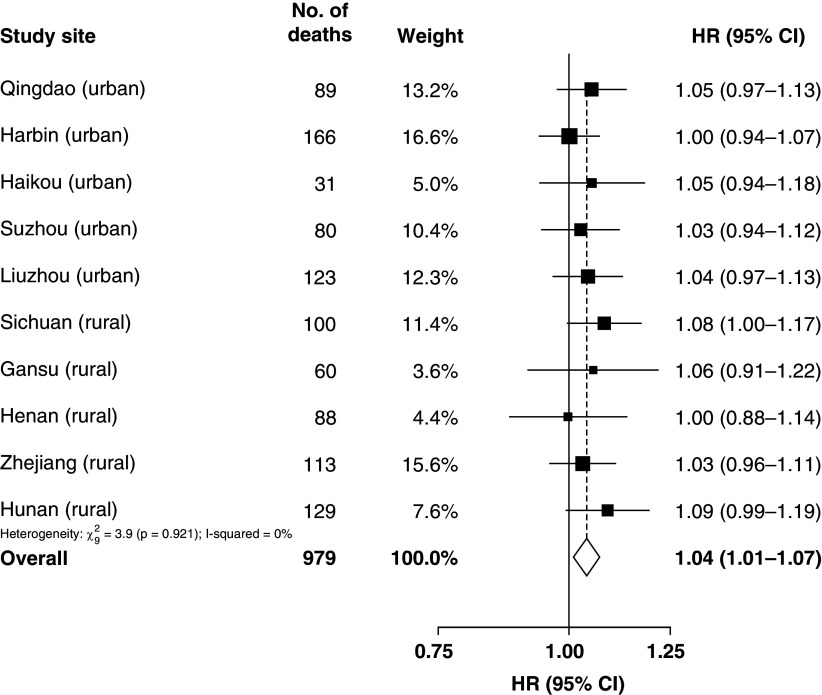
Region-specific association of household air pollution with lung cancer deaths among never-smokers and meta-analysis estimate in China Kadoorie Biobank (2004–2008). Hazard ratios (HRs) were calculated from multivariable analyses with baseline age as the underlying time variable and adjusted for sex, height, highest education, occupation, stove ventilation, solid fuel use for heating, physical activity, body mass index, diabetes, and intake of dairy products, using cooking oil use as a stratified variable. CI = confidence interval.

**Figure 3.**
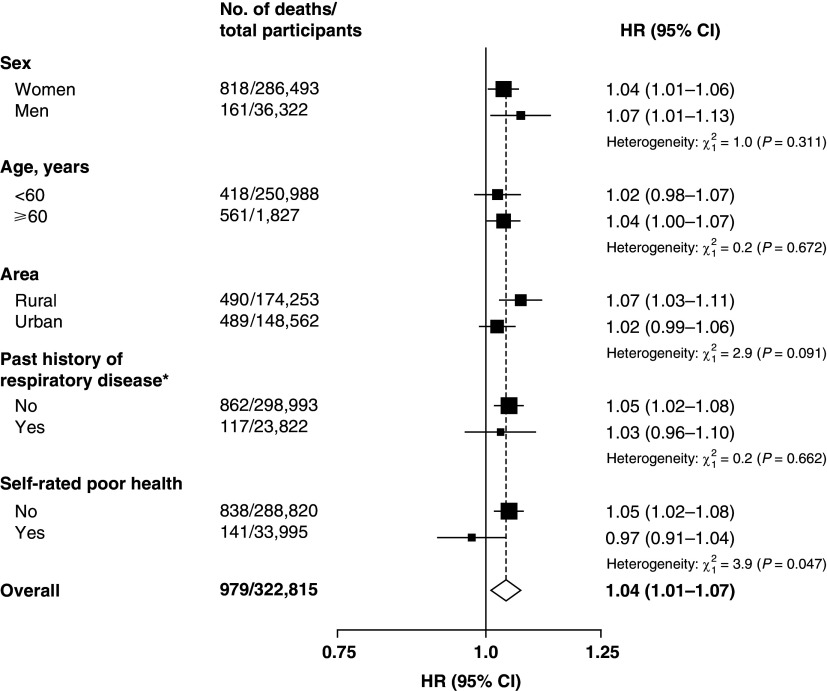
Subgroup analysis of lung cancer deaths among never-smokers in relation to household air pollution in China Kadoorie Biobank (2004–2008). HRs were calculated from multivariable analyses with baseline age as the underlying time variable and adjusted for sex, height, region of residence, highest education, occupation, stove ventilation, solid fuel use for heating, physical activity, body mass index, diabetes, and intake of dairy products, using cooking oil use as a stratified variable. CI = confidence interval; HR = hazard ratio. *Includes past history of tuberculosis, asthma, or chronic obstructive pulmonary disease.

During a median follow-up of 10.2 years (interquartile range, 9.3–11.2 yr), there were 979 lung cancer deaths (83.6% women). Ever exposure to HAP was not significantly associated with lung cancer death (HR, 1.15; 95% CI, 0.91–1.45; *P* = 0.26). Nevertheless, there was a significant log-linear positive association between cumulative duration of HAP exposure and lung cancer death (*P* trend = 0.018), with 4% increased risk per 5-year increment of exposure duration (HR, 1.04; 95% CI, 1.01–1.06) and no sign of departure from linearity (*P* = 0.12). When we excluded the group with >50 years of exposure, the *P* trend and *P* nonlinearity were 0.0034 and 0.33, respectively, in the range of 0–50 years of exposure (Figure 1). Notably, participants with 40.1–50.0 years (mean = 44.7 yr) of exposure had the highest risk compared with the never-exposed (HR, 1.53; 95% CI, 1.13–2.07), whereas those exposed for >50 years (mean, 62.1 yr) had a comparatively lower HR (1.27; 95% CI, 0.93–1.73) (*see* Table 2). Stratified analysis across 10 regions and the corresponding meta-analysis in Figure 2 showed largely consistent results (pooled HR per 5-year increment of exposure = 1.04; 95% CI, 1·01–1·07; I^2^ = 0%). Subgroup analyses in Figure 3 also found consistent results, though only a borderline significant difference between those who self-reported their health as “poor” and “not poor” (*P* heterogeneity = 0.047). The first sensitivity analysis using the longest duration of exposure among the three sources, and the second sensitivity analysis where data in the first 5 years of follow-up were excluded, showed a lung cancer risk increase per 5-year increment of exposure at 3% (HR, 1.03; 95% CI, 1.00–1.06; *P* = 0.027) and 5% (HR, 1.05; 95% CI, 1.01–1.09; *P* = 0.0082) respectively. In the second sensitivity analysis, 7,291 participants (including 468 who died of lung cancer) were excluded, with negligible missing values. For SHS, no evidence for association with lung cancer death was found for each of the four exposure variables in the multivariable analyses (Table 2). The interaction between HAP and SHS was not significant (*P* interaction = 0.23). The only covariate that violated the proportional hazards assumption was use of cooking oil (*P* = 0.04; all other covariates had a *P* value of more than 0.05). Model fit was improved when HAP was analyzed with cooking oil use as a stratified variable. Subsequently, the results were updated according to the final models of stratified analysis, and they were almost identical as the original analyses.

**Table 2.** Adjusted Hazard Ratios for Lung Cancer Deaths among Never-Smokers in Relation to Exposure to Household Air Pollution and Secondhand Tobacco Smoke in China Kadoorie Biobank (2004–2008)

	Lung Cancer Deaths		Multivariable Model*	
Exposures	Yes (%) *n* = *979*	No (%) *n* = *322,815*	HR (95% CI)	*P* Value^†^
Sex, *n* (%)				
Men	161 (16.5)	36,322 (11.3)	—	—
Women	818 (83.5)	286,493 (88.7)	—	—
Exposure to household air pollution				
Status of ever exposure, *n* (%)				
Never	96 (9.8)	49,140 (15.2)	1.00 (Reference)	0.250
Ever	883 (90.2)	273,675 (84.8)	1.15 (0.91–1.45)	—
Duration exposed, mean (SD), yr	31.2 (21.3)	24.4 (19.2)	—	—
Duration exposed, per unit change of 5 yr	—	—	1.04 (1.01–1.06)	0.009
Duration exposed, *n* (%), yr				
0	96 (9.8)	49,140 (15.2)	1.00 (Reference)	0.018 (trend)
0–10	105 (10.7)	39,652 (12.3)	1.14 (0.86–1.51)	—
10–20	118 (12.1)	59,276 (18.4)	0.95 (0.72–1.25)	—
20–30	168 (17.2)	58,965 (18.3)	1.26 (0.96–1.65)	—
30–40	155 (15.8)	49,135 (15.2)	1.34 (1.01–1.79)	—
40–50	161 (16.5)	34,515 (10.7)	1.53 (1.14–2.07)	—
>50	176 (18.0)	32,132 (10.0)	1.27 (0.93–1.74)	—
Exposure to secondhand smoke				
Lived with a person who smoked for >6 mo				
Never	230 (23.5)	65,726 (20.4)	1.00 (Reference)	0.710
Past	284 (29.0)	79,971 (24.8)	0.93 (0.77–1.11)	—
Current	465 (47.5)	177,118 (54.9)	0.96 (0.81–1.14)	—
Duration of living with a person who smoked among those who reported current exposure at baseline^‡^, mean (SD), yr	38.7 (17.7)	34.3 (15.2)	1.00 (1.00–1.01)	0.580
Status of ever exposure, *n* (%)				
Never	124 (12.7)	29,121 (9.0)	1.00 (Reference)	0.460
Ever	855 (87.3)	293,694 (91.0)	0.93 (0.76–1.13)	—
Duration of living with a person who smoked for >6 mo and frequency/duration of exposure at baseline				
Never lived with a person who smoked and never or occasionally exposed at baseline	190 (19.4)	52,243 (16.2)	1.00 (Reference)	0.520
Either never lived with a person who smoked or never or occasionally exposed at baseline	229 (23.4)	67,030 (20.8)	1.07 (0.88–1.30)	—
Ever lived with a person who smoked for ⩽30 yr and exposed at least 1 d/wk at baseline	160 (16.3)	80,621 (25.0)	0.93 (0.75–1.16)	—
Ever lived with a person who smoked for >30 yr and exposed <7 h/wk at baseline	261 (26.7)	68,809 (21.3)	0.98 (0.80–1.19)	—
Ever lived with a person who smoked for >30 yr and exposed ⩾7 h/wk at baseline	139 (14.2)	54,112 (16.8)	0.89 (0.71–1.12)	—

*Definition of abbreviations*: CI = confidence interval; HR = hazard ratio.

* Multivariable model with age as the underlying time variable and adjusted for sex, height, region of residence, highest education, occupation, stove ventilation, solid fuel use for heating, use of cooking oil, physical activity, body mass index, diabetes, and intake of dairy products.

^†^ Type 3 test for Cox regression.

^‡^ Confined to the participants who reported currently living with a person who smoked at baseline (177,583); among them, 465 had the outcome of lung cancer death.

## Discussion

To the best of our knowledge, this is one of the first and largest prospective cohort studies (with 323,794 never-smokers) to date to report association between HAP from domestic solid fuel use and lung cancer in never-smokers, and one of the first to evaluate the associations of exposure to HAP and SHS with lung cancer mortality among never-smokers in the same population. Our results suggest that HAP exposure was positively associated with lung cancer death in a dose–response manner with a 4% increased risk per 5-year increment of exposure duration. Although the association was not statistically significant in subgroups by regions (except Sichuan), the associations were largely consistent across the 10 regions (*P* heterogeneity = 0.921; I^2^ = 0%), and in various subgroups of participants defined by key personal characteristics, including the subgroups by sex and age. Notably, we found a null association between “ever exposed to HAP” (vs. never exposed) and lung cancer death, but this crude exposure classification has oversimplified the widely varying exposure time, and this would have decreased the precision of the risk estimates. Coherently, our results suggest that the elevated risk of lung cancer death was more apparent among participants with prolonged exposure to HAP with a statistically significant increased risk for those with at least 30 years’ exposure. The observed smaller HR for lung cancer death in participants exposed to HAP for more than 50 years (HR, 1.27; 95% CI, 0.93–1.73) was likely owing to survival and healthy volunteer bias, whereby individuals with extremely long exposure are less likely to be recruited into the study owing to poor health (if HAP has adverse health effect), or the relatively healthy individuals who joined the study with such exposure may be less vulnerable to the harm of HAP. No significant association was found between SHS and lung cancer death in our analyses, and no evidence of a statistical interaction between HAP and SHS was found, noting a very small number of participants were not exposed to both HAP and SHS.

This study provides new prospective evidence in a contemporary and geographically diverse study sample with a large number of active long-term solid fuel users, revealing a positive log-linear exposure–response relationship of years of HAP exposure with lung cancer death among never-smokers. Previous evidence on the association of solid fuel use with lung cancer risk was primarily from case-control studies, with substantial heterogeneity (25–27). One meta-analysis (2012) of 28 case-control studies with 12,419 cases and 34,609 controls reported that solid fuel use was associated with lung cancer risk, with a pooled odds ratio (OR) of 1.82 (95% CI, 1.60–2.06) for coal use and 1.50 (95% CI, 1.17–1.94) for biomass fuel use (25). Another meta-analysis (2015) examined 14 case-control studies with 8,221 cases and 11,342 controls, and with a focus on lung cancer risk associated with domestic biomass use, reported a lower pooled OR (1.17; 95% CI, 1.01–1.37) (27). Similarly, the two IARC monographs evaluating HAP (8, 9) relied largely on case-control studies that examined different types of solid fuels used by both ever- and never-smokers to establish an association between domestic solid fuel use and lung cancer risk. Most of these studies are limited by their retrospective nature, which incurs difficulty in establishing temporality, inevitable recall bias, as well as risk of residual confounding from smoking and SHS exposure, all of which may lead to an overestimated relative risk (RR).

In the two crucial cohort studies (11, 12) that supported IARC’s conclusions, RR estimates were back calculated from indirect evidence showing a significant reduction in lung cancer incidence or mortality associated with improved ventilation among lifetime solid fuel users. Consequently, there is considerable risk of bias from the nonrandomized adoption of ventilated cookstoves and nonadjustment for SHS, as well as bias due to unmeasured confounding factors, whereby increased socioeconomic status, improved air quality, and/or other government policies on energy use might have occurred concomitantly. A seminal retrospective cohort study in Xuanwei, China, also reported substantially higher lung cancer mortality among smoky-coal users than smokeless coal users for both men and women (10). These findings have been supported by several other studies (28, 29). However, as these previous cohort studies were conducted mostly among people born before the 1960s in a special area of China (Xuanwei, Yunnan Province) where lung cancer mortality rates and smoky-coal use prevalence are among the highest worldwide (30), the time period covered by these studies means that their RR estimates may not be entirely comparable to more modern cohorts, as the chemical component of commercial coal, the design and burning efficiency of domestic stoves, the background risk of lung cancer, and lung cancer treatment and survival have changed over time. More recently, the Shanghai Women’s Health Study prospective cohort reported a null association between coal use and lung cancer even with adjustment for ventilation, yet in a subgroup analysis for women with poor ventilation, a significant association with lung cancer risk was found for ever use of coal (HR, 1.69; 95% CI, 1.22–2.35) or ⩾20 years of coal use (HR, 2.03; 95% CI, 1.35–3.05) compared with never exposure. However, the significant findings were limited to small samples of women (*n* = 5,357–10,840; 38–67 lung cancer cases) residing in one geographical location (31), where only 1% of participants were persistent coal users (32).

The exact underlying mechanisms for the carcinogenicity of solid fuels remain unclear. It is, however, known that domestic combustion of solid fuels generates a large amount of particulate matter (PM) (33), a mixture of hundreds of noxious chemicals including high concentrations of heterocyclic aromatic compounds and polycyclic aromatic hydrocarbons, many of which are confirmed or suspected human carcinogens (34) that (when activated) can bind covalently to DNA to form stable or depurinating adducts, and induce oxidative damage (35). Epidemiological evidence suggested that a methylated PAH, 5-methylchrysene, found experimentally as the major class of mutagens in combustion products of smoky coal in Xuanwei, could be a potential driver of lung cancer (36). Other studies also reported that each 10-mg/m^3^ increase in PM ⩽2.5 μm in aerodynamic diameter concentrations was associated with a 15–27% increase in lung cancer mortality (37), and long-term exposure to air pollutants and high genetic risk synergistically increased the risk of lung cancer (38).

SHS was classified by the IARC in 2004 and 2012 as a group 1 agent (9, 39) (i.e., “human carcinogen with sufficient evidence for lung cancer development”), with evidence supported by both case-control and cohort studies. However, findings from the cohort studies were rather inconsistent (13–16). In a recent meta-analysis of 41 studies of nonsmoking women in Asia, Europe, and North America, Ni and colleagues (2018) found a significant association between SHS and lung cancer in 34 case-control studies (pooled OR, 1.35; 95% CI, 1.17–1.56), but the association did not reach statistical significance in the seven cohort studies (pooled RR, 1.17; 95% CI, 0.94–1.44) (40). The discrepancy between case-control and cohort studies warrants reflection on the limitations owing to small sample size and potential recall bias. Interestingly, we also found no statistically significant prospective association between SHS and lung cancer death in this study, regardless of the SHS indicators used, a result that is consistent with findings from some previous cohort studies (14, 15). Nonetheless, it is important to note that reliable assessment of SHS exposure is a major challenge in virtually all existing studies, including the present investigation, as it can be affected by a complex array of factors, including the intensity and proximity of smoking, underreported exposure in the reference group, and potential confounding due to occupational or other environmental factors (39). It is also worth noting that 93.9% of our participants had ever been exposed to SHS, which reflects the generally high smoking prevalence and relatively low degree of SHS control in China until recently. The relatively limited heterogeneity in SHS exposure in the CKB population may have masked the true association with lung cancer mortality, so further investigation in younger cohorts who would have experienced more drastic differences in SHS exposure owing to the increasingly stringent SHS control policies may help to clarify this.

This study has several strengths. In addition to the large sample size and prospective design with a relatively long follow-up period, our study covered a wide range of risk factors of interest, with satisfactory reproducibility on a comprehensive range of information (18). Also, our data covered diverse geographical regions with different amounts of HAP exposure, and the assessment of exposure to HAP and SHS in relation to lung cancer mortality among never-smokers in the same population allowed assessment of potential interaction between HAP and SHS. Also, results from sensitivity analyses suggest that our observed risk estimates are robust. Furthermore, although we used mortality data instead of incidence data in this study, the wider coverage of mortality data in China suggests that it is potentially a preferred option.

This study has some limitations, too. First, exposures to HAP and SHS were measured using self-reported proxies (fuel use and lived with people who smoked), which could lead to exposure misclassification and thus dilution of association due to random measurement error (41). Nevertheless, the reproducibility of reported HAP exposure was acceptable, as indicated by the consistent responses between the baseline survey and resurvey (Table E3) (23). Smoking status was also self-reported, so some never-smokers might actually be light, occasional, or long-term ex-smokers. Validation from measured exhaled carbon monoxide in another CKB study (42) suggests little contamination from current smoking at baseline, but some ex-smokers may still have been classified as never-smokers. Second, only the primary cooking and heating type was assessed by self-reporting, leaving measurement bias owing to historical or concurrent exposure to solid fuels emissions from secondary fuels or solid fuel smoke in the neighborhood. This, along with regional variability in using different types of solid fuels (coal vs. wood), has hindered our ability to explore the potentially different associations with coal versus wood implicated in previous studies (10, 31). However, it is also important to note that most previous studies on coal focused on bituminous coal, the usage of which is highly regionally dependent, and CKB regions are not known to rely on bituminous coal. Third, despite restricting the analyses to never-smokers and extensive adjustment for potential confounders, residual confounding from other known or unknown risk factors for lung cancer that were not ascertained (e.g., occupational exposure to fumes) cannot be ruled out. Fourth, as the resulting cumulative duration of HAP exposure was the sum of the aggregated duration of residence with cooking solid fuel use, stove smoky-coal use, and coal-smoky home in winters, there was a possible overestimation of the actual duration of exposure. Because the duration of exposure is strongly correlated with age, we cannot rule out residual confounding by age. Nevertheless, as there were consistent findings from the first sensitivity analysis in which the longest duration of exposure among the three sources was used for analysis, it indicates that the impact was likely to be minimal.

Furthermore, we were unable to assess the impact of HAP and/or SHS exposure within specific periods of life on lung cancer death, such as during childhood, given the pervasive extent of exposure across the life-course for most participants. Also, owing to the lack of detailed direct exposure measurement data (e.g., PM ⩽2.5 μm in aerodynamic diameter) across seasons, the seasonal nature of heating fuel use may be fully and directly accounted for when deriving overall cumulative HAP exposure, especially given the correlation between heating and cooking fuel use. To address this, we attempted to adjust the regional differences in seasonal variation of indoor heating by applying region-specific weighting coefficients derived from ambient temperature variations. Lastly, given the linkage between HAP and several other major causes of death, competing risk of death from other causes is possible, which may result in an underestimation of the independent association between HAP and lung cancer risk. Future investigations with more comprehensive follow-up data on other causes of death are warranted to clarify this.

## Conclusions

This large-scale prospective study provides valuable evidence suggesting that duration of exposure to domestic solid fuel use is associated with increased lung cancer death in a dose–response manner among never-smokers in China, with consistent results across 10 Chinese regions and in subgroups of participants defined by several key personal characteristics. It adds to the evidence base on the potential carcinogenicity of domestic solid fuels used for cooking and heating in many developing countries and rural regions across the world (43). Our findings highlight the potential lung cancer burden associated with HAP in addition to those from other major respiratory and cardiovascular diseases (20, 44) and reinforce the need for public health organizations and policymakers to promote strategies to reduce HAP exposure (4). Our findings on SHS call for further studies, ideally with better assessment of SHS exposure, to add large-scale prospective evidence for evaluation by the IARC Working Group.
